# Anticardiolipin Autoantibodies as Useful Biomarkers for the Prediction of Mortality in Septic Patients

**DOI:** 10.1155/2022/9775111

**Published:** 2022-05-31

**Authors:** Amal Abouda, Z. Hajjej, A. Mansart, W. Kaabechi, D. Elhaj Mahmoud, O. Lamine, E. Ghazouani, M. Ferjani, I. Labbene

**Affiliations:** ^1^Intensive Care Unit, Military Hospital of Tunis, Tunisia; ^2^Laboratory of Immunology, Military Hospital of Tunis, Tunisia; ^3^Research Laboratory LR12DN01, Tunis, Tunisia; ^4^University of Tunis El Manar, Tunisia; ^5^University of Paris-Saclay, UVSQ, INSERM U1173, 2I, France; ^6^FHU SEPSIS (Saclay and Paris Seine Nord Endeavour to PerSonalize Interventions for Sepsis), Garches, France; ^7^RHU RECORDS (Rapid Recognition of Corticosteroid Resistant or Sensitive Sepsis), Garches, France; ^8^Medicine Faculty of Tunis, Department of Basic Sciences, Tunis El Manar University, Tunis, Tunisia; ^9^Immuno-Rheumatology Research Laboratory, Rheumatology Department, La Rabta Hospital, University of Tunis-El Manar, Tunis, Tunisia; ^10^Department of Biochemistry, Military Hospital of Tunis, Montfleury, Tunis, Tunisia

## Abstract

**Background:**

The detection of antiphospholipid antibodies (aPL) is of interest because of their importance in the pathogenesis of arterial or venous thrombosis. They could be a “second hit” of an inflammatory event such as infection. The aim of our study was to assess the performance of antiphospholipid antibody biomarker to predict in-hospital mortality in intensive care unit (ICU) septic patients.

**Methods:**

We conducted a prospective single-center observational study including consecutive critically ill septic adults admitted to the intensive care unit. Clinical and laboratory data including enzyme-linked immunosorbent assay for antiphospholipid antibodies (anticardiolipin (aCL), antiphosphatidylserine (aPS)) were obtained. Blood samples were collected on days 1, 3, 5, 8, and 10 of hospitalization. The primary study endpoint was ICU mortality defined as death before ICU discharge. Secondary end points included correlation between SOFA score and biological parameters.

**Results:**

A total of 53 patients were enrolled. 18.8% of patients were aPL positive. In-hospital mortality rate was 60%. Multivariate analysis showed that age and aCL at days 3 and 5 along with SOFA at day 3 were independent outcome predictors. A significant positive correlation existed between SOFA at days 3, 5, and 8 and antiphospholipid antibody concentrations.

**Conclusions:**

Our data showed that antiphospholipid was useful biomarkers for the prediction of mortality in critically ill septic patients. We found a positive correlation between SOFA score and antiphospholipid antibodies.

## 1. Introduction

Sepsis is one of the oldest syndromes in clinical medicine [1]. The definition of sepsis has been revised, in 2016, declaring sepsis as a life-threatening organ dysfunction triggered by a dysregulated host response to infection [2]. According to the third international consensus for sepsis and septic shock (sepsis-3), organ dysfunctions can be characterized by an increase in the score of two or more points in the sequential Sepsis-related Organ Failure Assessment (SOFA) correlated with in-hospital mortality over 10% [2]. Following these updates, a growing understanding of the physiopathology of this disease has been realized [3]. However, the mortality linked to this syndrome remains high [4].

Recently, new endogenous actors have enriched the diagnosis of sepsis and septic shock, acting as danger signals provided by the response to infection [5–7], including antiphospholipid antibodies (aPL) [8]. aPL is a member of a heterogeneous family of autoantibodies acting against membrane phospholipids or antiphospholipid-binding proteins [9]. The development of antiphospholipid antibodies during bacterial, viral, or parasitic infections [10–12] is a common and most frequently temporary.

These antibodies are responsible for a wide variety of clinical events, such as thrombosis which may lead to the development of dysfunction, and are associated with poor short and long-term prognosis in critically ill patients [13]. Anticardiolipin is one of these antibodies implicated in bacterial and viral sepsis [14, 15] as well as antiphosphatidylserine, which is implicated in immunothrombosis as reported [16].

The presence of APL antibodies may be useful as a second-line test for optimizing thrombotic risk stratification. However, the role of aPL as a serologic marker is debated. In this study, we tried to determine the diagnostic and clinical importance of the aPL in sepsis-3, illustrating the incidence of aCL and aPS in sepsis-3, which might be useful in prognostic. We hypothesize that aPL production, even transitory, is associated with increased duration of organ failure measured by SOFA score and could be used as an indicator to judge the severity of sepsis.

Moreover, we have tried to assess the performance of antiphospholipid antibody biomarker to predict in-hospital mortality in intensive care unit (ICU) septic patients.

## 2. Methods

### 2.1. Patients and Study Design

The study was designed as a prospective, controlled, clinical trial and performed in an 18-bed medical surgical intensive care unit at Tunis military hospital (Tunisia).

From January 2019 to December 2019, all patients, admitted to the ICU for sepsis and older than 18 years, were eligible for this study.

Exclusion criteria were pregnancy, clinical history of antiphospholipid syndrome or auto-immune diseases, and immunosuppressive treatment.

The study was reviewed and approved by the Institutional Ethics Authorities (26/2019/CLPP/Hôpital Militaire de Tunis) and was registered with Clinicaltrials.gov, number NCT04685278.

### 2.2. Data Collection

The following information was retrieved: gender, age,, Sequential Organ Failure Assessment (SOFA) score [17], reasons for hospitalization, etiological diagnosis, worst and best vital signs during the ICU stay, comorbidities, ICU length of stay (LOS), source of infection, causal organisms, use of antimicrobials, and clinical ICU outcomes. The SOFA score was determined at the time of ICU admission then daily. Laboratory variables were retrieved from the database specific to laboratorial data.

### 2.3. Sampling and Measurement of aCL and aPS

Blood samples were collected in dry tubes on days 1, 3, 5, 8, and 10 of hospitalization. Blood samples had been centrifuged at 2000 rpm for 5 minutes. Serum was collected and aliquoted in Eppendorf tubes and then preserved at -20°C for subsequent assays.

The enzyme-linked immunoassay, indirect-type ELISA, was applied for the aCL and aPS highlighting to the three types of immunoglobulins IgM, IgG, and IgA by commercial combined isotypes ELISA kits (EUROIMMUN®, Germany). The normal value was ≤12 RU/mL for all isotypes. Accordingly, patients having a concentration of aCL and/or aPS > 12 RU/mL at any time of the study were considered as aPL positive patients; otherwise, they were negative.

### 2.4. Study Endpoints

The primary study endpoint was ICU mortality defined as death before ICU discharge. Secondary end points included correlation between SOFA score and antiphospholipid antibodies.

### 2.5. Statistical Analysis

The SPSS v.22.0 (Armonk, USA) software was used for data analysis. Continuous variables are expressed as mean ± standard deviation (SD), while categorical variables are expressed with absolute and relative frequencies. The normality assumption of continuous variables was evaluated using the Kolmogorove Smirnov criterion. Univariate comparisons (survivors vs. nonsurvivors) were made using either two-sample Student's *t*-tests or Wilcoxon rank sum tests for the continuous variables and either chi-square tests or Fisher's exact tests for the categorical variables. Two multiple logistic regression analyses were performed with dependent variables those defined from univariate analyses, using stepwise backward elimination with a significance level for removal of *p* ≤ 0.10 in order to find the best model fitting our data. All reported *p* values are two-tailed. Statistical significance was set at *p* < 0.05.

Graphics were sketched using Prism 5 (GraphPad software, USA).

## 3. Results

### 3.1. Demographic Data

A total of 53 patients, 40 men and 13 women, were enrolled over a one-year period. The predominant sepsis origins were pulmonary and urinary tract followed by skin and soft tissue. The in-hospital mortality rate was 60%.

In univariate comparisons, there was a statistically significant difference between survivors and nonsurvivors for age, SOFA score at days 3 and 5, diabetes mellitus as comorbidities, and septic shock incidence but not in thrombotic events (including pulmonary embolism, thrombi, disseminated intravascular coagulation, and deep vein thrombosis) (Table 1).

### 3.2. Prevalence and Association of aPL with In-Hospital Mortality

Based on the manufacturer's cut-off, 10 out of 53 patients (18.8%) were aPL positive.

**Figure 1:**
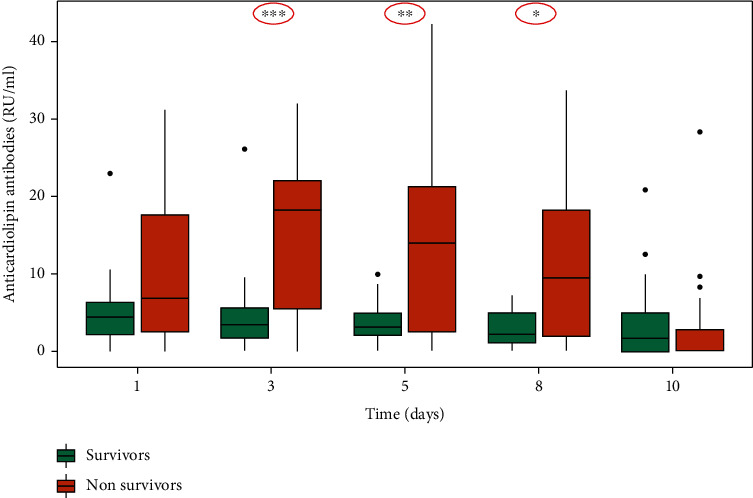
Association of anticardiolipin antibodies (aCL) with in-hospital mortality.

We found an association between aCL and hospital mortality with a statistically significant difference at day 3 (*p* = 0.0001), day 5 (*p* = 0.002), and day 8 in the study (*p* = 0.03) (Figure 1).

**Figure 2:**
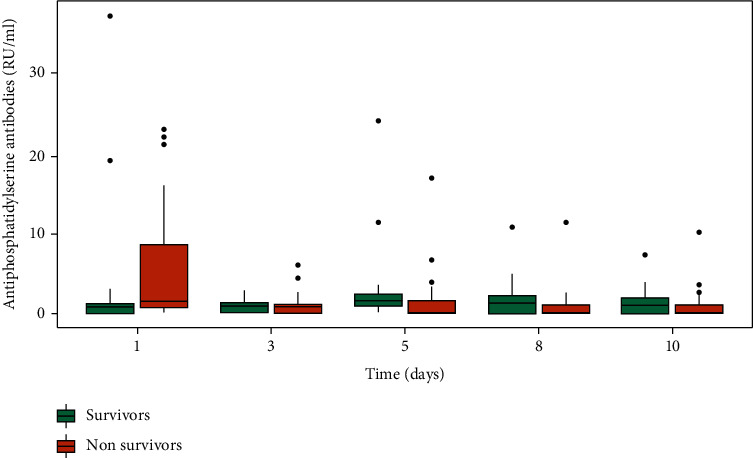
Association of antiphosphatidylserine antibodies (aPS) with in-hospital mortality.

There was no significant difference between survivors and nonsurvivors for aPS (Figure 2).

Multivariate logistic regression modeling was then performed. We found that aCL production was significantly associated with higher mortality at day 3 (OR: 3.457 (0.08-0.290); *p* = 0.01) and at day 5 (OR: 1.815 (0.01-0.560); *p* = 0.04). Age and SOFA score at day 3 were also significantly associated with high mortality (OR: 3.595 (0.04-0.340) and *p* = 0.01 and OR: 1.208 (0.102-0.992) and *p* = 0.033, respectively) (Table 2).

### 3.3. aPL and SOFA Score Correlation

**Figure 3:**
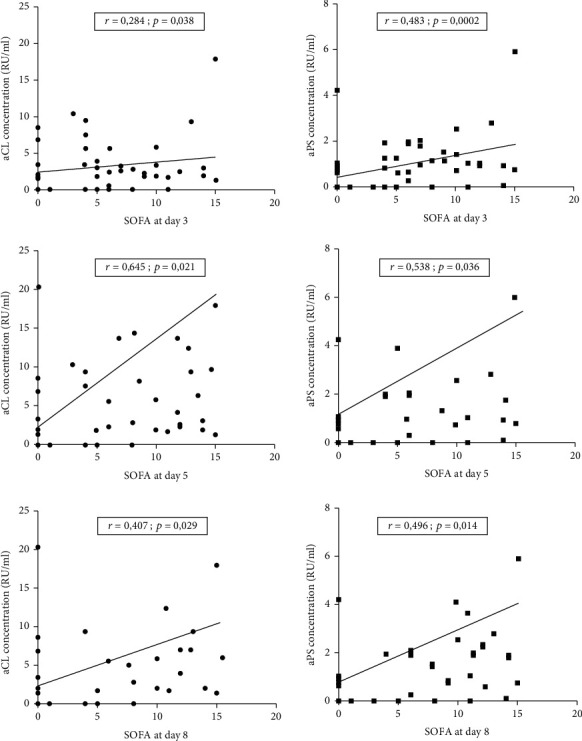
Correlation between SOFA score and aPL antibodies.

Significant positive linear correlations were found between serum levels of both aCL and aPS and SOFA score from day 3 (*p* = 0.038; *p* = 0.002), day 5 (*p* = 0.021; *p* = 0.036) to day 8 (*p* = 0.029; *p* = 0.014), respectively, but not at day 1 and day 10 (Figure 3).

### 3.4. Evolution of SOFA Score among Positive aPL Patients

**Figure 4:**
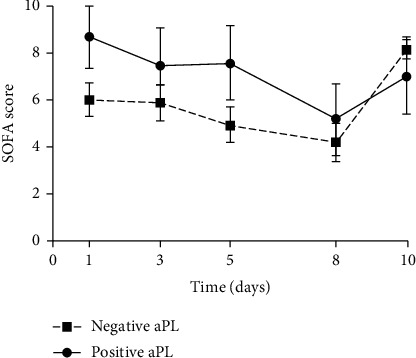
Evolution of SOFA score among aPL positive and aPL negative groups.

A higher SOFA score was observed in aPL positive patients as compared to aPL negative ones. However, this difference did not reach statistical significance (Figure 4).

### 3.5. Correlation between aPL with Thrombotic and Inflammatory Markers

We detected that only the aPS level was correlated with platelets (*p* = 0.002) and prothrombin (*p* = 0.038) at day 5. In addition, levels of CRP and aPL correlate, starting from day 5.

From day 5 to day 10, aCL correlate with CRP at the same time of measurement (*r* = 0.431, *p* = 0.002; *r* = 0.620, *p* < 0.0001; *r* = 0.756, *p* < 0.0001) and the same for aPS (*r* = 0.508, *p* < 0.0001; *r* = 0.699, *p* < 0.0001; *r* = 0.746, *p* < 0.0001).

**Figure 5:**
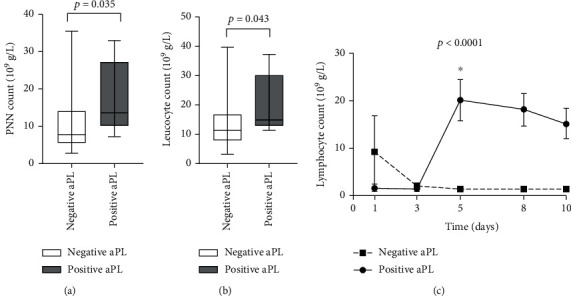
(a) Polynuclear neutrophil (PNN) cell count at day 5. (b) Leucocyte cell count at day 5. (c) Evolution of lymphocyte count from day 1 to day 10 in positive and negative aPL patients. Data are means ± SEM.

Thereafter, we raised the question whether the presence of aPL influences the immune cell count. Essentially, we verified absolute polynuclear neutrophil (PNN) count and lymphocyte as main contributors. Polynuclear neutrophils, leucocytes, and lymphocytes were higher in positive aPL patients compared to negative aPL patients, from day 5, as shown in Figure 5.

Furthermore, we tested possible associations between aPL, PNN, and lymphocytes. We found that the absolute polynuclear neutrophil (PNN) count at day 5, significantly, correlates with aCL titer at day 8 (*r* = 0.358, *p* = 0.034).

## 4. Discussion

Sepsis is a disastrous highly complex disease condition, with significant contributors to the host immune responses and inflammation [17, 18]. Leading cause of mortality in patients admitted to ICU, this pathology is hence a major public health concern [19, 20]. Exploring new biomarkers for early detection of sepsis risk and disease control would improve the prognosis of patients.

Knowing that sepsis causes dynamic changes in the coagulation system that occur in both bleeding and thrombosis [21] and that aPL autoantibodies play a significant role in thrombotic events [22], its causal relationship with infections becomes of interest but remains insufficiently explored [10]. Our results illustrated some aspects of these complex changes in sepsis, and we hypothesized that aPL might have the potential for predicting future organ failure and could be used as an indicator to predict in-hospital mortality in intensive care unit (ICU). Our findings are supported by previous studies which reported the occurrence of aPL as pathogenic in different diseases such as in cancer [23, 24], acute respiratory distress syndrome (ARDS) [25], human immunodeficiency virus (HIV) [26], acute kidney injury (AKI) [27], and more recently in COVID-19 [11, 28–30]. In our study, we measured aCL IgM, IgG, IgA and aPS IgM, IgG, and IgA autoantibodies in septic patients, without a clinical history of auto-immune disease, at different time points during their hospitalization in ICU. These autoantibodies may occur in critically ill patients following different infections [8, 28]. We detected that in the current study, 18.8% of the patients were aPL positive, which is not uncommon. This frequency is in accordance with the prevalence reported in the study of Kalgudi and Ho, in which 18% of the patients had raised IgG or IgM aCL titers after severe traumatic brain injury [31]. The high prevalence of aPL in septic patients may be the consequence of the high risk of exposition to exogenic antigens by means of infected medical instruments [32] or nosocomial infections. Other researchers demonstrated that aPL antibodies were associated with hemodialysis vascular access thrombosis in 19.8% of hemodialysis patients [33].

However, in other diseases such as COVID-19, the prevalence of aPL was more variable. In fact, a prospective observational study performed in Madrid found that only 8.3% of the patients were positive with anticardiolipin IgM and anti-*β*2-glycoproteinI IgM [34]. In contrast, Zuo et al. found that 52% of their patients proved positive for aPL in patients with SARS-CoV-2 [28]. The hypercoagulable state and the high rate of thrombosis, of all these patients, were common.

In our study, this increase in aPL production was associated to the in-hospital mortality. More importantly, these results showed that the measure of aCL at day 3 and day 5 was associated to in-hospital mortality. Moreover, in this study, aCL, rather than aPS, measurement at day 3 was an early marker of SOFA score. The aCL as aPS levels showed a positive correlation with SOFA score, on the same days of different measurements, at day 5 and at day 8 of sepsis diagnosis.

Our results are in line with Riancho-Zarrabeitia et al. who found that antiphospholipid syndrome in patients with systemic lupus erythematosus predicts a more serious disease with higher accrual damage and higher mortality rates [35].

We, also, detected that only the aPS level was correlated with platelets and prothrombin at day 5. This result could be explained by the overexpression of phosphatidylserine (PS) at the surface of activated platelets. These activated platelets play a significant role in fibrin and thrombin production. By translocating from the internal to the external leaflet of the membrane, PS aids in the formation of the intrinsic tenase complex (factor (F) VIIIa; FIXa; FX) and the prothrombinase complex (FVa; FXa; prothrombin) during the propagation phase of coagulation [36].

Moreover, levels of CRP and aPL correlate at different time points of the study starting from day 5, showing the implication of aPL in the inflammatory process; for example, we observed an elevation in leucocytes, polynuclear neutrophils, and lymphocyte count at day 5 for aPL positive patients.

In the present analysis, some limitations need to be discussed. A critical first step in any observational study is the number of patients, which can be increased. Then, it is difficult to compare clinical studies, because multiple research designs are used with a wide range of aPL assays. There is currently no gold standard for the detection of APL antibodies. Solid step ELISA approaches are highly efficient, and many quality control systems have shown that aPL tests generate a substantial interlaboratory variation in outcomes.

Despite the shortcomings detected in this study, we speculate that the association between aPL production and mortality rate in patients with sepsis can be considered as a promising result, since we assessed the time of this production. As it may be a relevant biomarker, the development of aPL in response to therapy is of clinical significance. Interestingly, we revealed, for the first time, that aPL may be used as an independent indicator to predict mortality in critically ill patients, to stratify and assess the prognosis of infectious diseases such as sepsis.

## Figures and Tables

**Table 1:** Demographic characteristics of the investigated patients.

	Total (*n* = 53)	Survivor (*n* = 21)	Nonsurvivor (*n* = 32)	*p*
Age, years (mean ± SD)	47.1 ± 17.3	39.6 ± 20.7	64.2 ± 13.9	0.004
Male (*n*, %)	40 (75%)	15 (71%)	25 (78%)	0.307
Comorbidities (*n*, %)				
Diabetes mellitus	19 (35.8%)	4 (19%)	15 (46%)	0.022
Dyslipidemia	14 (26.4%)	6 (28.5%)	8 (25%)	0.412
Hypertension	14 (26.4%)	6 (28.5%)	8 (25%)	0.361
Chronic pulmonary disease	6 (11.3%)	2 (9.5%)	4 (12.5%)	0.284
Chronic renal failure	2 (3.7%)	1 (4.7%)	1 (3.1%)	0.521
Sepsis origin (*n*, %)				
Pulmonary	31 (58.4%)	14 (66.5%)	17 (53%)	0.491
Abdominal	1 (1.8%)	1 (4.7%)	0 (0%)	
Central venous catheter	3 (5.6%)	1 (4.7%)	2 (6.2%)	
Urinary tract	7 (13.2%)	3 (14.2%)	4 (12.5%)	
Skin and soft tissue	6 (11.3%)	2 (9.5%)	4 (12.5%)	
Nonidentified	5 (9.4%)	2 (9.5%)	3 (9.3%)	
Septic shock (*n*, %)	29 (54.7%)	7 (33%)	22 (69%)	0.012
Thrombotic events (*n*, %)	11 (20.75%)	7 (33%)	4 (12.5%)	0.067
Length of stay	23.13 ± 53	17.81 ± 15.7	26.74 ± 67.7	0.081
SOFA (mean ± SD)				
Baseline	16.1 ± 9.1	14.8 ± 6.1	15.1 ± 7.1	0.642
Day 1	15.3 ± 7.9	10.2 ± 3.9	14.7 ± 7.3	0.221
Day 3	10.1 ± 4.5	7.1 ± 2.5	13.3 ± 6.4	0.001
Day 5	7.3 ± 3.9	4.3 ± 1.9	10.8 ± 5.4	0.002
Day 8	8.4 ± 2.1	8.1 ± 2.7	9.1 ± 5.8	0.364
Day 10	7.9 ± 4.6	6.3 ± 1.1	8 ± 3.2	0.210
Mortality (%)	60			

SOFA: Sequential Organ Failure Assessment score; SD: standard deviation.

**Table 2:** Variables associated with mortality in multivariate regression modeling.

Variable	OR (95% CI)	*p*
Age	3.595 (0.04–0.340)	0.01
Diabetes mellitus	1.014 (0.830-1.237)	0.316
SOFA		
Day 3	1.208 (0.102-0.992)	0.033
Day 5	0.146 (0.052–1.032)	0.106
Septic shock	0.584 (0.165–1.289)	0.584
Length of stay	0.688 (0.361–1.462)	0.609
Anticardiolipin antibodies		
Day 3	3.457 (0.08–0.290)	0.01
Day 5	1.815 (0.01–0.560)	0.04
Day 8	1.398 (0.08–1.061)	0.169

OR: odds ratio; CI: confidence interval.

## Data Availability

Data are available on request.

## Abbreviations

aPL: Antiphospholipid
aCL: Anticardiolipin
aPS: Antiphosphatidylserine
SOFA: Sepsis-related Organ Failure Assessment
ICU: Intensive care unit.
